# Inactivation of miR-34a by aberrant CpG methylation in Kazakh patients with esophageal carcinoma

**DOI:** 10.1186/1756-9966-33-20

**Published:** 2014-02-17

**Authors:** Xiaobin Cui, Zhimin Zhao, Dong Liu, Tao Guo, Su Li, Jianming Hu, Chunxia Liu, Lan Yang, Yuwen Cao, Jinfang Jiang, Weihua Liang, Wei Liu, Shugang Li, Lianghai Wang, Lidong Wang, Wenyi Gu, Chuanyue Wu, Yunzhao Chen, Feng Li

**Affiliations:** 1 Department of Pathology and Key Laboratory for Xinjiang Endemic and Ethnic Diseases, Shihezi University School of Medicine, North 4th Road, Shihezi, Xinjiang 832002, China; 2 Department of Oncology, Tongji Hospital, Huazhong University of Science and Technology, Wuhan, Hubei 430030, China; 3 Henan Key Laboratory for Esophageal Cancer, Zhengzhou University School of Medicine, Zhengzhou, Henan 450001, China; 4 Australian Institute for Bioengineering and Nanotechnology, The University of Queensland, Brisbane QLD 4072, Australia; 5 Department of Pathology, University of Pittsburgh, Pittsburgh, PA 15261, USA

**Keywords:** MiR-34a, Esophageal squamous cell carcinoma, Kazakh, Methylation

## Abstract

**Background:**

Esophageal squamous cell carcinoma (ESCC) is an aggressive tumor with dismal prognosis and high incidence and mortality in Kazakh population. MiR-34a, a direct p53 target gene, possesses tumor-suppressive properties as they mediate apoptosis, cell cycle arrest, and senescence. The reduced expression of miR-34a by methylation in various cancers has been reported.

**Methods:**

To determine whether aberrant miR-34a methylation occurs in esophageal cancer, the DNA methylation of 23 CpGs sites in the miR-34a promoter was quantitatively analyzed in relation to the translation initiation site by MALDI -TOF mass spectrometry in 59 ESCC tissues and 34 normal tissues from the Kazakh population. Real-time PCR was used to detect the inhibition of miR-34a expression levels and to evaluate their association with methylation.

**Results:**

We found that miR-34a is more frequently methylated in ESCC (0.133 ± 0.040) than in controls (0.066 ± 0.045, P < 0.01). A nearly two-fold increase in miR-34a expression for the hypomethylated promoter was found in normal esophageal tissues than ESCC with hypermethylation (P <0.0001), pointing to a negative relationship between miR-34a CpG sites methylation and expression(r = −0.594, P = 0.042). The hypermethylation of miR-34a CpG_8.9 was associated with the advanced UICC stage III/IV of the esophageal cancers, and the hypermethylation of CpG_8.9 and CpG_5 of miR-34a was significantly correlated with lymph node metastasis.

**Conclusions:**

Our findings suggest that miR-34a is involved in the etiology of ESCC and that hypermethylated miR-34a is a potential biomarker for ESCC diagnosis and prognosis. Moreover, targeting miR-34a methylation by demethylating agents may offer a novel strategy for anticancer therapy of ESCC.

## Introduction

Esophageal squamous cell carcinoma (ESCC) is one of the most malignant cancers worldwide, ranking as the fourth most common cause of cancer-related deaths in China [1]. Compared with other ethnic populations in China and those in Xinjiang, where most Chinese Kazakhs reside, the Kazakh population is characterized by higher incidence and mortality (90-150/100 000, age standardized) of ESCC than those in the general population of China [2-4]. Genetic defects, including the mutation or amplification of oncogenes and the inhibition of tumor-suppressor genes, such as EGFR, KRAS, pRb, and cyclin D1 mutations [5-8], are involved in the carcinogenesis of ESCC. In addition, it is well established that p53 mutation is the most common genetic alteration in 60.6% of ESCC [9]. By contrast, gene methylation is an alternative mechanism of gene inactivation that occurs early tumor progression and thus alters gene expression without changing the DNA sequence [10-12]. Similar to genetic mutations, transcriptional silencing by CpG methylation is stably inherited to the next cell generation and may therefore allow the clonal expansion of a cell population with a selective advantage during tumor progression. Various tumor-suppressor genes that regulate apoptosis, the cell cycle, and cell signaling are aberrantly methylated in ESCC [12-14].Given these observations, uncovering the molecular pathogenesis of Kazakh ESCC, especially the detection of aberrant CpG methylation, is therefore likely to provide new approaches to the prevention, diagnosis and treatment of ESCC.

MicroRNA (miRNA), a class of small regulatory RNA molecules, acts as tumor suppressors and oncogenes by negatively regulating their mRNA targets in a sequence-specific manner through post-transcriptional repression and influencing the proliferation and cell cycle progression, apoptosis, invasion and metastasis of cancer [10]. Widespread miRNA is dysregulated in various human malignancies by changes in DNA copy number and epigenetic inactivation, although their exact functions during carcinogenesis are still being examined [15-17]. In esophageal cancer, the reduced expression of miR-143 or the overexpression of miR-7 is reportedly correlated with the depth of invasion and lymph node metastasis of ESCC [18]. Among the types of miRNAs, the miR-34a gene, which resides in chromosome 1q36.22 and belongs to the miR-34 family, reportedly is directly regulated by the p53 transcription factor [19,20]. The miR-34a downregulates numerous important regulatory proteins of cell cycle progression and apoptosis, such as E2F3, c-MYC, Bcl2, c-MET, and CDK4/6, suggesting that miR-34a itself may mediate tumor suppression [21]. The reduced or absent expression of miR-34a was reported in 110 cancer cells lines, such as breast, lung, colon, kidney, melanoma, bladder, pancreatic carcinoma, lymphoma, and myeloma and cell lines, and two different types of primary cancers (melanoma and primary neuroblastoma samples) because of the aberrant CpG methylation of its promoter [22-24]. However, only one study have reported that the miR-34a was silenced in ESCC cell lines and re-expression miR-34a can inhibit the ESCC proliferation by reducing the C-met and Cyclin D1 expression [24], yet the correlation between downregulation/loss of miR-34a expression and promoter methylation in ESCC was not clean, especially in the Kazakh population.

Given that aberrant DNA methylation is an important mechanism for gene transcription and protein expression silencing, in the present study, we accordingly therefore hypothesized whether epigenetic modifications indirectly modulate miR-34a expression by silencing or activating miR-34a genes in Kazakh ESCC patients. To address this problem, using the matrix-assisted laser desorption ionization time-of-flight mass spectrometry (MALDI-TOF MS) approach, we quantitatively evaluated the individual CpG unit methylation in 318 base pairs regions in length (proximal region encompassing the transcription start site and the p53 binding sites) containing 23 CpG sites within 15 CpG units at the miR-34a promoter regions with a total of 93 Kazakh subjects. The relationship between the promoter methylation and gene expression of miR-34a in patients with and without ESCC in additional samples was also examined to explore the mechanism of the development of Kazakh ESCC. The promoter hypermethylation of the miR-34a gene was correlated with the downregulation of mRNA expression in Kazakh ESCC, providing insight into the molecular mechanism of Kazakh esophageal cancer and the pathogenesis of the cancer in relation to the function of the hypermethylation of the miR-34a promoter.

## Materials and methods

### Patients and tissue samples

Fifty-nine esophageal tissues from Kazakh patients diagnosed with histologically confirmed ESCC were randomly collected by multistage cluster sampling. All patients were recruited from the First Affiliated Hospital of Shihezi University and the People’s Hospital of Xinjiang Uygur Autonomous Region between 1984 and 2011. No restrictions regarding age, sex, or disease stage were set. Patients who had undergone surgery (other than diagnostic biopsies), chemotherapy, or radiation therapy before recruitment or any blood transfusion in the preceding six months were excluded. All samples were surgically resected, fixed in 10% buffered formalin, routinely processed, and embedded in paraffin. We gathered data on clinic-pathological variables, such as tumor site, invasion depth, and distant metastasis from the medical records of the patients. The differentiation grade, TNM stage, and lymph node status were classified according to the UICC/AJCC TNM classification (seventh edition). For comparison, 34 samples of normal esophageal tissue were obtained from materials surgically resected from 34 patients without any primary esophageal tumor.

In this study, various clinic-pathological characteristics of Kazakh ESCC cases and controls were investigated as follows (Additional file 1: Table S1). The age was 55.1 ± 8.26 (mean ± SD) years for the cancer samples and 44.7 ± 7.8 (mean ± SD) years for the normal sample (P =0.54). There were 32 (54.2%) males and 27 (45.8%) females in the case group and 19 (55.9%) males and 15 (44.1%) females in the control group (P = 0.87). The cases included 14 (23.7%) well-differentiated patients (group G1), 30 (50.9%) moderately differentiated patients (G2), and 15 (25.4%) poorly differentiated patients (G3). Of the 59 ESCC cases, 32 (54.2%) were classified as stage I/II and 27 (45.8%) as stage III/IV. Thirty-three of the patients presented with lymph node metastases.

This study was approved by the Research Ethics Committee of Shihezi University School of Medicine, P. R. China. Written informed consent was obtained from all of the patients. All specimens were handled and made anonymous according to the ethical and legal standards.

### DNA isolation and bisulfate conversion

DNA was isolated from 10 tissue sections of 10 μm thickness by proteinase K digestion and a tissue DNA extraction kit (Qiagen Inc., Valencia, CA, USA) according to the manufacturer’s protocol. As an internal control, all purified genomic DNA samples were successfully tested by polymerase chain reaction (PCR) with human β-actin primers For: 5′-CAGACACCATGGTGCACCTGAC-3′ and Rev: 5′-CCAATAGGCAGAGAGAGTCAGTG-3′, indicating that the suitable quality and quantity of DNA can be used to detect the profile of miR-34a methylation. Genomic DNA was stored at −20°C until use as a template for each PCR reaction. The genomic DNA was treated with bisulfite through an EZ DNA Methylation KitTM according to the manufacturer’s instructions (Zymo Research, Orange, CA, USA) (Catalog No. D5001). This treatment combines bisulfate conversion and DNA clean-up. The converted DNA was measured by an ND-1000 spectrophotometer (NanoDrop Technologies, Inc., Wilmington, DE, USA).

### Quantitative analysis of DNA methylation

**Figure 1:**
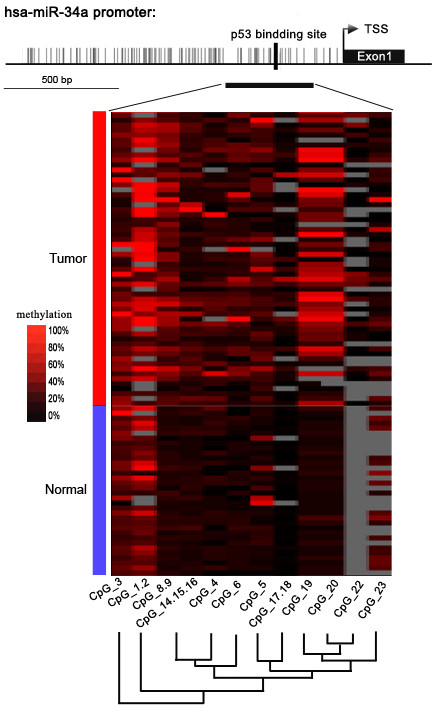
**Genomic structure of distribution of miR-34a CpG dinucleotides over transcription start site (TSS) and hierarchical cluster analysis of CpG units’ methylation profiles of miR-34a promoter region in tumor (*n*** **= 59) and normal (*n*** **= 34) tissues.** The depicted region corresponds to 1.2 kbp upstream of the TSS (indicated by arrow). Each vertex indicates an individual CpG site. The positions and orientation of the MassARRAY primers are indicated by horizontal black bars. The position of the p53 binding site is indicated. Columns display the clustering of CpG units, which are a single CpG site or a combination of CpG sites. Each row represents a sample. The methylation intensity of each miR-34a CpG unit in each sample varies from red to black, which represents high to low expression. The color gradient between black and red indicates methylation ranging from 0 to 100. Gray represents technically inadequate or missing data.

The sequence of the CpG island was identified by the UCSC genome browser (http://genome.ucsc.edu/). Given that the genomic region upstream the p53 binding site in the miR-34a gene revealed a prominent CpG island, we selected the area with proximal promoter activity in previous experiments [22]. The analyzed region and the CpG sites of the miR-34a promoter are shown in Figure 1. We designed primer sets for the methylation analysis of the miR-34a promoter region by EpiDesigner software (http://epidesigner.com; Table 1). For each reverse primer, an additional T7 promoter tag was added for in vivo transcription, and a 10-mer tag was added to the forward primer to adjust for differences in melting temperature. The DNA methylation of miR-34a was quantitatively analyzed by the MassARRAY platform (SEQUENOM) as previously described [25]. The 5 μl PCR mixture contained 10 ng of bisulfite-treated DNA, 25 mM dNTP, 0.2 U of Hot Start TaqDNA polymerase (Sequenom, Sequenom Inc., San Diego, CA, USA), and a 1 μM mixture of forward and reverse primers. The cycles included pre-heating at 94°C for 4 min, followed by incubation for 45 cycles of 94°C for 20 s, 62°C for 30 s, and 72°C for 60 s and then by incubation at 72°C for 3 min. Two microliters of a shrimp alkaline phosphatase (SAP) mix containing 1.7 μl of H2O and 0.3 μl (1.7 U) of SAP (Sequenom) was added to digest redundant dNTPs with the following program: 37°C for 20 min, 85°C for 5 min, and 4°C thereafter. After the SAP treatment, unincorporated dNTPs were dephosphorylated by adding 2 ml of premix including 0.3 U of SAP (Sequenom). The reaction mixture was incubated at 37°C for 40 min, and the SAP was heat-inactivated for 5 min at 85°C and was then maintained at 4°C. Five microliters of T Cleavage Transcription/RNase Cocktail including 0.89 μl of 5× T7 polymerase buffer, 0.24 μl of T cleavage mix, 3.14 mM dithiothreitol, 22 U of T7 RNA and DNA polymerase, 0.09 mg/ml of RNase A, and 2 μl of the product of the PCR/SAP reactions was mixed and incubated under the following conditions: 37°C for 3 h of in vitro transcription and RNase A digestion. Fifteen nanoliters of cleavage reaction was then robotically dispensed (by a nanodispenser) onto silicon chips preloaded with a matrix (SpectroCHIP; SEQUENOM, San Diego). Mass spectra were collected by MassARRAY Compact MALDI-TOF (SEQUENOM), and the methylation proportions of the spectra were generated by Epityper 1.0 software (SEQUENOM, San Diego). All the experiments were performed in triplicate. Inapplicable readings and their corresponding sites were eliminated from analysis. The methylation level was expressed as the percentage of methylated cytosines over the total number of methylated and unmethylated cytosines.

**Table 1:** Sequences of PCR primers used in this study

**Gene**	**Primer**	**Sequence(5′-3′)**	**Product size (bp)**
miR-34a	tag-FW	5′ -aggaagagagGTTTATTTGGGTGTATGTTGGGA-3′	318
	T7-RV	5′-cagtaatacgactcactatagggagaaggctACCTAATCCTCTTTCCTTTTCAAAT-3′	
β-globin	For	5′-CAGACACCATGGTGCACCTGAC-3′	210
	Rev	5′-CCAATAGGCAGAGAGAGTCAGTG-3′	

“FW”: Forward, “RV”: Reverse.

### cDNA synthesis and real-time PCR

Real-time PCR was conducted in two steps as previously described. RNA was extracted from ESCC cells with the RNeasy Mini Kit (Qiagen, Hilden, Germany). cDNA was amplified with specific primer sets: MiR-34a (Hs_miR-34a_1 miScript Primer Assay, MS00003318) and RNU6 (Hs_RNU6-2_1 miScript Primer Assay, MS00033740) in a Stratagene Mx-3000P real-time thermocycler (Stratagene, La Jolla, CA). Quantitative real-time PCR was conducted with a SYBR green PCR Master Mix (Qiagen, Hilden, Germany) containing ROX as a reference dye. Data were normalized for RNU6 (housekeeping gene) expression by the comparative threshold cycle method. Triplicate *C*_*t*_ values were averaged, and the relative expression levels of the four ESCC cell lines were determined as 2^−∆Ct^ (∆C_t_ = C_t miR-34a in ESCC tissues_ − C_t RNU6 gene in normal tissues_).

### Statistical analysis

Data were analyzed in GraphPad Prism 5.0 (GraphPad Software Inc., San Diego, CA, USA) and SPSS 13.0 (SPSS Inc., Chicago, IL, USA). All P values were two-sided, and the significance level was P < 0.05. A Mann–Whitney U-test was performed to compare the miR-34a methylation levels of every CpG site between the ESCC and control groups and between male and female subjects. The association between each CpG site methylation of miR-34a and the clinicopathologic parameters was evaluated by a nonparametric test (the Mann–Whitney U-test between two groups and the Kruskal–Wallis H test for three or more groups). Spearman correlation was analyzed to evaluate the correlations between the CpG site methylation level of miR-34a and its expression levels. Two-sample t-tests were conducted to compare the miR-34a expression between ESCC and normal tissues.

## Results

### Hypermethylation of miR-34a promoter in Kazakh patients with ESCC

The MassARRAY system is a tool for the high-throughput detection and quantitative analysis of methylation at a single CpG site at a target fragment (CpG island) that generates accurate data that represent the ratio or frequency of methylation events on a CpG site by MALDI-TOF MS. This system was used to assess the methylation profile of miR-34a in all the samples collected from Kazakh patients with ESCC (n =59) and from control subjects (n = 34). The amplicon detected in the promoter regions of miR-34a was 318 base pairs in length (proximal region encompassing the transcription start site and the p53 binding sites) and contained 23 CpG sites that can be divided into 15 CpG units.

**Figure 2:**
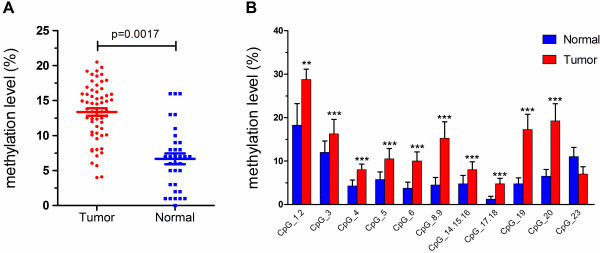
**Evaluation of promoter methylation of miR-34a. (A)** Comparison of average methylation status of miR-34a promoter between control and ESCC subjects. **(B)** Median methylation levels of 11 informative CpG units in miR-34a promoter between control and ESCC subjects. **P < 0.01, ***P < 0.001 (Mann–Whitney U-test).

Among these CpG units, four CpG units (7 CpG sites) yield unsuccessful measurements. The final dataset consisted of 11 CpG units (2,139 sites in 93 analyzed samples), and the individual CpG unit methylation of miR-34a that distinguished ESCC from normal tissues is depicted in the cluster diagram (Figure 1). The patterns observed in the cluster analyses show that the methylation status of normal controls was notably different from that observed in tumor tissues. The overall methylation level of the target fragment of the miR-34a promoter was statistically higher (0.133 ± 0.040) in Kazakh esophageal cancer than in normal tissues (0.066 ± 0.045, *P* < 0.01, Figure 2A). The methylation level of every CpG unit within the miR-34a promoter was also evaluated (Figure 2B). Apart from that CpG_23, the mean methylation levels at CpG_1.2, CpG_3, CpG_4, CpG_5, CpG_6, CpG_8.9, CpG_14.15.16, CpG_17.18, CpG_19 and CpG_20 were all significantly higher in patients with ESCC (mean methylation = 28.75%, 16.25%, 8.00%, 10.50%, 10.00%, 15.25%, 8.00%, 4.75%, 17.25%, 19.25%, respectively) than in the controls(mean methylation = 18.25%, 12.00%, 4.25%, 5.75%, 3.75%, 4.50%, 4.75%, 1.25%, 4.75%, 6.50%, respectively; all the P values are less than 0.01).

### Hypermethylated miR-34a in esophageal carcinoma is associated with metastasis development

**Figure 3:**
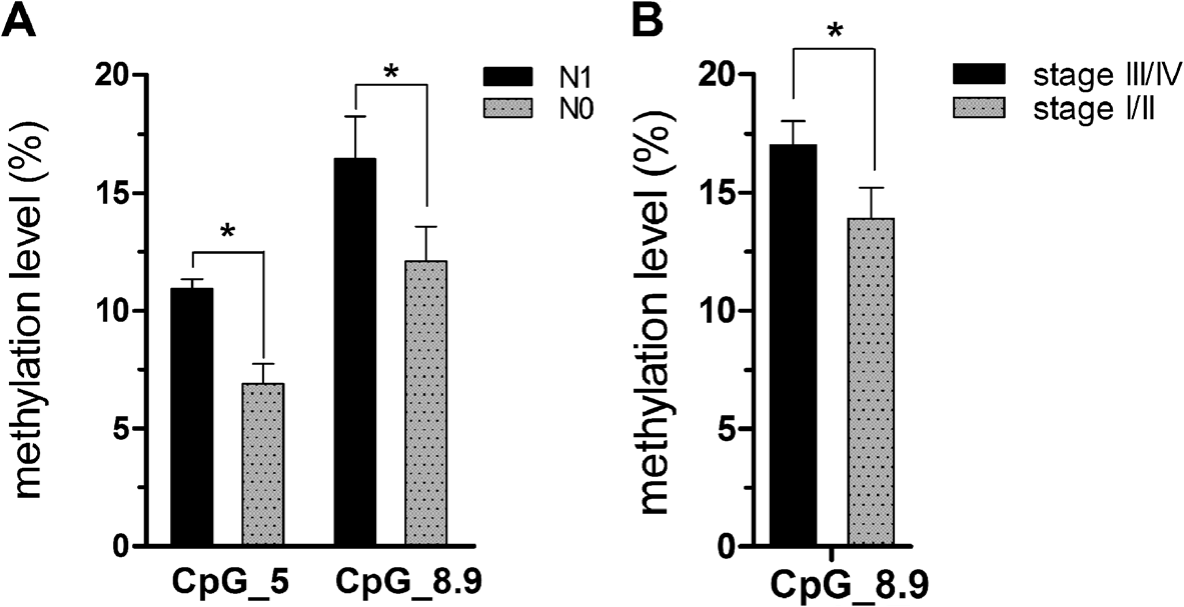
**Association between miR-34a methylation level and clinicopathologic features in ESCC patients (Mann–Whitney U-test). (A)** Tumors with lymph node metastasis (N1) and without (N0). **(B)** TNM stage. *P < 0.05.

The association between the patterns of the quantitative methylation of every CpG unit within the miR-34a promoter and the clinicopathologic features of the 59 Kazakh patients with ESCC was further evaluated (Table 2). The CpG_5 and CpG_8.9 methylation levels of miR-34a in lymph node metastasis tumor tissue were remarkably greater than those in tumor tissue without lymph node metastasis (10.9% vs. 6.9%, p = 0.026; 16.4% vs. 12.1%, p = 0.022, respectively; two-tailed Mann–Whitney U-test). The CpG_8.9 methylation levels of miR-34a in tumor-stage III/IV tissues were also significantly higher than those stage I/II tissues (17.0% vs. 13.9%, P = 0.029; two-tailed Mann–Whitney U-test, Figure 3). However, no correlation was found between the other CpG units methylation of miR-34a and age at diagnosis, gender, and tumor differentiation of Kazakh ESCC.

**Table 2:** Association between miR-34a promoter methylation and clinicopathologic features in ESCC patients

**CpG unit**	**CpG site**	**Clinical characteristic (Z/P)**											
		**Gender**^¶^		**Age**^¶^		**Tumor location**^¶^		**Differentiation**^**#**^		**Lymphatic metastasis**^¶^		**TNM stage**^¶^	
Unit1	CpG_1.2	−1.396	0.163	−0.364	0.716	−1.227	0.220	0.334	0.846	−0.628	0.530	−0.838	0.402
Unit2	CpG_3	−1.075	0.282	−0.259	0.796	−1.592	0.057	5.813	0.055	−0.397	0.691	−1.440	0.150
Unit3	CpG_4	−1.558	0.119	−0.457	0.648	−1.359	0.174	2.136	0.344	−0.708	0.479	−1.019	0.308
Unit4	CpG_5	−0.039	0.969	−0.528	0.598	−0.607	0.544	1.901	0.386	−2.223	0.026*	−0.625	0.532
Unit5	CpG_6	−0.168	0.866	−0.330	0.741	−1.057	0.291	2.992	0.224	−1.551	0.121	−0.732	0.464
Unit7	CpG_8.9	−0.450	0.653	−0.076	0.939	−0.093	0.926	2.221	0.896	−2.299	0.022*	−2.188	0.029*
Unit9	CpG_14.15.16	−1.429	0.153	−0.360	0.719	−0.891	0.373	1.940	0.379	−0.029	0.976	−0.092	0.926
Unit10	CpG_17.18	−0.086	0.931	−0.770	0.441	−0.160	0.873	2.183	0.336	−0.612	0.541	−4.70	0.638
Unit11	CpG_19	−0.211	0.833	−0.459	0.646	−0.397	0.691	0.225	0.893	−0.328	0.743	−0.967	0.334
Unit12	CpG_20	−0.382	0.702	−0.692	0.489	−0.559	0.576	0.137	0.934	−0.328	0.743	−1.077	0.282
Unit15	CpG_23	−0.128	0.898	−0.460	0.646	−1.696	0.090	0.735	0.692	−0.711	0.477	−0.174	0.862

Note: ^¶^Mann–Whitney U test (two-sided); ^**#**^Kruskal-Wallis H test (two-sided); *P < 0.05, bold face representing significant data.

### Suppresion of miR-34a in Kazakh ESCC tissue

**Figure 4:**
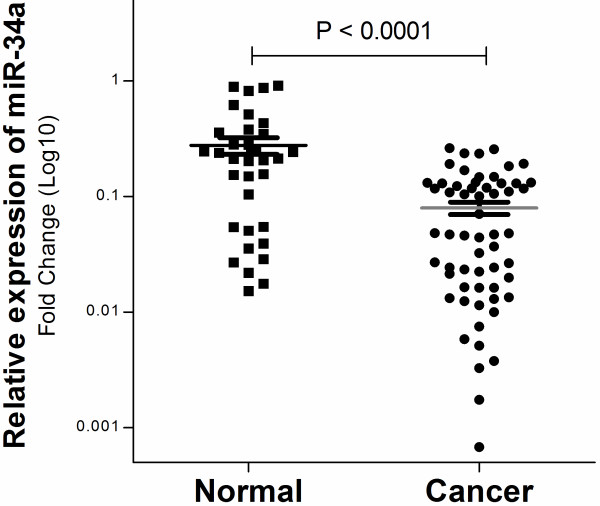
**Average relative miR-34a expression level in ESCC compared with that in normal esophageal tissues.** The expression level of miR-34a was measured by qRT-PCR and was normalized by U6RNA. Each sample was analyzed in triplicate, repeated three times. Error bars represent the standard error of mean, and asterisks represent a statistically significant difference (P < 0.0001).

To determine whether CpG methylation is accompanied by decreased miR-34a expression, we examined expression of miR-34a mRNA by real-time PCR in the same cohort (tumor n = 59; normal n = 34) used for the methylation analysis. The results, consistent with our expectation, indicated that the miR-34a gene showed a nearly two-fold decrease in expression in Kazakh ESCC patients with a high level of methylation compared with that in normal tissues (0.079 ± 0.094 vs. 0.277 ± 0.045, P < 0.0001; Figure 4).

### Correlation between promoter methylation and expression of miR-34a

**Figure 5:**
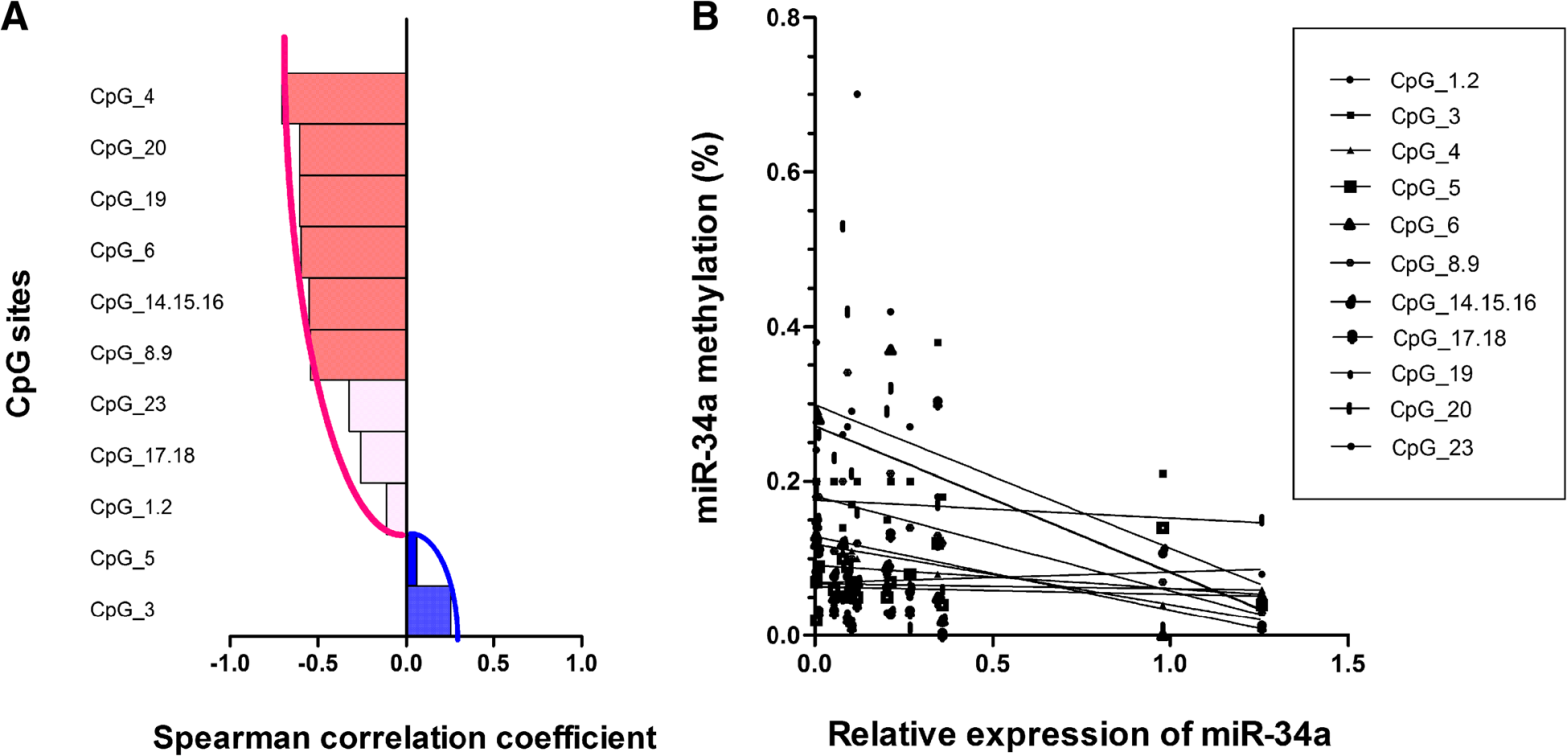
**Negative correlation of miR-34a specific CpG units’ methylation and their expression. (A)** Bar plot of Spearman correlation coefficient (r) showing strength of negative correlation between miR-34a expression and methylation value of each CpG unit within miR-34a, with negative values representing inverse correlations and positive values representing positive correlations. Significant correlations (P < 0.05) are indicated in red. **(B)** Analysis of scatterplots and simple linear regression graphically displaying the correlation between methylation level of each CpG unit and miR-34a gene expression in Kazakh ESCC samples by Spearman correlation coefficient analysis. The straight line was the “best fit” that indicated the trend of relationship.

We analyzed the Spearman correlation between the methylation levels at individual CpG units and their expression. This analysis yielded 11 correlation coefficients [range: (−0.705) to (+0.263)] (Figure 5A). Notably, a significant inverse correlation was observed for CpG_4, CpG_6, CpG_8.9, CpG_14.15.16, CpG_19, and CpG_20 methylation and miR-34a expression (Figure 5B and Table 3). A negative relationship between global miR-34a methylation and mRNA expression was also observed in relation to the overall methylation status of the miR-34a promoter and gene expression (*r* = −0.594, P = 0.042). These results demonstrated that the hypermethylation of the miR-34a promoter region might be the reason for the suppression of mRNA in Kazakh ESCC tissues.

**Table 3:** Correlation analysis of DNA methylation of individual CpG sites and miR-34a mRNA expression in Kazakh ESCC patients

**CpG unit**	**CpG site**	**Spearman’s correlation coefficient**	**P value**
Unit1	CpG_1.2	−0.113	0.713
Unit2	CpG_3	0.253	0.363
Unit3	CpG_4	−0.705	0.005
Unit4	CpG_5	0.059	0.834
Unit5	CpG_6	−0.597	0.019
Unit7	CpG_8.9	−0.545	0.036
Unit9	CpG_14.15.16	−0.552	0.033
Unit10	CpG_17.18	−0.259	0.372
Unit11	CpG_19	−0.606	0.017
Unit12	CpG_20	−0.606	0.017
Unit15	CpG_23	−0.324	0.28

P values correspond to two-sided Spearman correlation tests.

## Discussion

MiRNAs is an important regulator of protein post-transcriptional regulation in a sequence-specific manner. MiR-34a is the direct transcriptional targets of p53. As members of the p53 regulation network, miR-34a induces apoptosis and a cell cycle arrest in the G1-phase and targets Notch, HMGA2, and Bcl-2 genes involved in the self-renewal and survival of cancer stem cells, thereby suppressing tumor cell proliferation, which is dysregulated in many cancers [26]. MiR-34a is hypermethylated in non-small-cell lung cancer (64%, 20/31), melanoma (62.5%, 20/32), and prost ate carcinoma (79.1%, 19/24) [22,27]. In contrast to the regulation of other miRNAs, miR-34a regulation in esophageal cancer is only partially understood. Studies of the methylation levels of the region 100 to 500 base-pairs upstream of the miR-34a transcription start, which includes the p53 binding site, in the prostate and pancreas carcinoma cell lines, such as LNCaP, PC-3, LAPC-4 and TsuPr1, have shown a significant correlation between the silencing of miR-34a expression and the levels of CpG methylation of the region 400 base-pairs promoter region of the miR-34a, which includes the p53 binding site [22]. In the present study, we examined the same region in the esophageal tissues and quantitatively detected the methylation patter by MALDI -TOF mass spectrometry. The promoter region of the miR-34a gene was frequently methylated in esophageal cancer and its methylation was related to loss of miR-34a expression. These results suggest that aberrant promoter methylation plays an important role in the down-regulation of miR-34a gene expression in Kazakh patients with esophageal cancer.

DNA methylation acts as an important switch that controls gene expression in cancer where methylation exhibits tumor-specific patterns [10]. To date, various ESCC-susceptible genes with aberrant DNA methylation or gene expression have been identified, such as RASSF1A genes [13]. miRNAs considerablely affects the initiation and progression of human cancers and therefore represent promising targets for anticancer therapies. Patterns of aberrant miRNA expression are involved in ESCC, and miRNA acts as oncogenes or tumor suppressors [28,29]. In the present study, we successfully replicated the results of the study by Chen *et al*. in the Chinese Han population by the traditional method [30], methylation-specific PCR (MSP), not the quantitative method, although the participants in both studies had different genetic and environmental backgrounds. The research conducted by Chen *et al*. have found that the methylation ratio of miR-34a is 66.7% (36/54) in ESCC patients from Chinese Han population, which are significantly higher than that in the corresponding non-tumor tissues [30]. However, previous studies have identified ethnic variations in DNA methylation levels related to lifestyle and dietary differences [31]. Consequence, with non-quantitative MSP method in Chinese Han population and the quantitative MassARRAY approach in Kazakh population, the uniformity of the methylation of the miR-34a promoter in both studies strengthens the association between such methylation and ESCC. Although miR-34a is epigenetically silenced in numerous cancers, including colorectal, pancreatic, mammary, ovarian, urothelial, renal cell carcinomas, and soft tissue sarcomas [22,32], the finding presented here is the first to demonstrate the suppression of miR-34a via promoter methylation in Kazakh patients with esophageal cancer.

Epidemiological and etiological studies have shown that the carcinogenesis and development of ESCC involves multiple factors and changes in gene expression [2,33-36]. Recent data suggest that dysregulation of miR-34a exists in various types of human cancers and is associated with clinic treatment [22,23,26,27,32,37,38]. Here, we found that miR-34a, direct transcriptional targets of the p53, showed a nearly two-fold elevated expression in normal esophageal tissues compared with that in tissues of Kazakh patients with esophageal cancer, in accordance with the results in a study by Hu [24]. Moreover, miR-34a mRNA expression is inversely correlated with the methyaltion of the miR-34a promoter, as reported by Chen et al., confirming the likely role of methylation in the regulation of miR-34a expression [30]. It is generally recognized that promoter methylation blocks transcription and mRNA expression by preventing binding of transcription factor. In our results, the promoter region of the miR-34a contains multiple CpG islands and sites [22], but the negative correlation between the quantitative hypermethylation level of each CpG sites and the expression was observed only in certain CpG sites. The results indicates that multiple CpG sites, and not methylation of every site down-regulated or suppressed gene expression. Only several CpG sites performed genetic transcription, and the methylated sites were the key CpG sites, perhaps the most remarkable finding of the present study.

Previous studies have demonstrated that miR-34a is a direct target of p53, our study revealed a novel mechanism for miR-34a regulation in Kazakh ESCC. Recently, there is growing evidence that p53 abnormality is not always associated with the down-regulation of miR-34a in human cancer tissues, although several groups have shown that the well-known tumour suppressive activity of p53 is at least in part moderated by miR-34a [19,20,39,40]. The expression of p53 resulted in up-regulation of miR-34a in the lung cancer cell line H1299 and the overexpression of miR-34a suppressed proliferation of lung cancer cells in vitro and promoted apoptosis [39]. Deletion or mutation of p53 is associated with miR-34a down-regulation in chronic lymphocytic leukemia and ovarian cancers [27,41,42]. While in neuroblastoma and small-cell lung cancer, no significant correlation between p53 mutation and miR-34a dysregulation is observed [43,44]. However, there was no direct correlation between the deletion or mutation of p53 and miR-34a expression levels in ESCC samples. Like other malignancies, mutations of p53 are common molecular genetic events in 60.6% of ESCC [9]. The observation of aberrant methylation of miR-34a-induced inactivation raises an important regulation mechanism for miR-34a in the etiology of Kazakh ESCC. It has been hypothesized that miR-34a promoter methylation preferentially occurs in tumors expressing mutant-type p53 in esophageal carcinoma. Clearly, future studies are required to obtain a more complete understanding of the consequence of miR-34a delivery to ESCC cells with mutant-type p53.

Our data show the significant correlation of two CpG sites’ methylation of miR-34a promoter with lymph node metastasis of Kazakh patients with esophageal carcinoma and thus suggest that miR-34a is an effective prognostic marker. This observation is in good agreement with the report that the methylation of miR-34 promoter is correlated with the metastatic potential of tumor cells, such as SIHN-011B, osteosarcoma and breast cancer cells lines [37,38,45], but not accordance with the results from Chen et al. [30]. Moreover, we analyzed the each CpG site’s methylation level of miR-34a and lymph node metastasis in esophageal carcinoma, but a significant correlation between them was observed only on two CpG sites, indicating that the overall methylation level cannot represent the clinical value. Therefore, only the accurate information of CpG sits’ methylation levels represents the clinical application value. However, the exact mechanism for the function of miR-34a epigenetic silencing in metastasis formation remains unambiguous. P53 was found to modulate miR-34a expression. Several studies have successfully discovered target genes of miR-34a involved the invasion and metastasis in many tumors. Molecularly, miR-34a suppresses breast cancer invasion and metastasis by directly targeting Fra-1 and inhibits the metastasis of osteosarcoma cells by repressing the expression of CD44 [37,38]. An ectopic expression of miR-34a in IMR90 cells substantially inhibits growth. However, no study on the miR-34a-targeted gene in ESCC has explained why miRNA promotes the metastasis. Therefore, the biological function of the higher rates of miR-34a promoter methylation in Kazakh ESCC should be further analyzed to clarify this point.

## Conclusions

Our findings not only for the first time demonstrate that miR-34a CpG island hypermethylation-mediated silencing of miR-34a with tumor suppressor features contributes to esophageal carcinoma in Kazakh population but also show that particular DNA methylation signatures of miR-34a CpG sites are associated with the metastatic of esophageal carcinoma. One application is that it is a potential methylation biomarker for the early diagnosis of esophageal carcinoma and the prediction of metastatic behavior. Most importantly, miR-34a may provide a mechanistic and molecular basis for the new therapeutic use of pharmacological compounds with DNA demethylating activity to treat Kazakh patients with esophageal carcinoma or metastatic development.

## Competing interests

The authors declare that they have no competing interests.

## Authors’ contributions

FL and YZC participated in the design of the study and coordination; XBC and ZMZ wrote the manuscript; XBC, ZMZ, and WL performed the MALDI -TOF mass spectrometry for miR-34a methylation. TG, YWC, LHW, JFJ and LY performed real-time PCR for quantification of miR-34a expression; DL, TG, SL, and JMH participated in recruitment of patients and collection and assembly of data; CXL, SGL and WHL performed statistical analysis; CYW and LDW helped to draft the manuscript and participated in the design of the study. All authors read and approved the final manuscript.

## Supplementary Material

Additional file 1: Table S1 The clinicopathological demographics for the 59 Kazakh patients with ESCC. Click here for file
