# Supplementary material for: Inactivation of miR-34a by aberrant CpG methylation in Kazakh patients with esophageal carcinoma
Source: J Exp Clin Cancer Res. 2014 Feb 17;33(1):20. doi: 10.1186/1756-9966-33-20 (PMC3931274; doi:10.1186/1756-9966-33-20)
Supplement: Additional file 1: Table S1 — The clinicopathological demographics for the 59 Kazakh patients with ESCC. [file 1756-9966-33-20-S1.doc]

**Supplementary Table 1 The Clinicopathological demographics for the 59 Kazakh patients with ESCC**

| **Patient characteristic** | **Number of patients (%)**  **(n=59)** |
| --- | --- |
| Gender  Male | 32(54.2) |
| Famale | 27(45.8) |
| Age(years) |  |
| <56 | 33(55.9) |
| ≥56 | 26(44.1) |
| Median | 55 |
| Range | 37-75 |
| Tumor location |  |
| Cervical | 41(69.5) |
| Thoracic | 18(30.5) |
| Differentiation ^1^ |  |
| G1 | 14(23.7) |
| G2-G3 | 45(76.3) |
| Lymphatic invasion |  |
| N0 | 26(44.1) |
| N1-N3 | 33(55.9) |
| TNM stage ^2^ |  |
| I/II | 32 (54.2) |
| III/IV | 27 (45.8) |

^1^ Histologic grade was with reference to WHO classification published in 2009.

^2^ TNM stage was based on the UICC criteria published in 2009.
